# Association of Statin Therapy With Major Adverse Cardiovascular and Limb Outcomes in Patients With End-stage Kidney Disease and Peripheral Artery Disease Receiving Maintenance Dialysis

**DOI:** 10.1001/jamanetworkopen.2022.29706

**Published:** 2022-09-01

**Authors:** Hao-Yun Lo, Yu-Sheng Lin, Donna Shu-Han Lin, Jen-Kuang Lee, Wen-Jone Chen

**Affiliations:** 1 Division of Cardiology, Department of Internal Medicine, National Taiwan University Hospital, Taipei, Taiwan; 2 Division of Cardiology, Department of Internal Medicine, Chang Gung Memorial Hospital, Chiayi, Taiwan; 3 College of Medicine, Graduate Institute of Clinical Medical Sciences, Chang Gung University, Taoyuan City, Taiwan; 4 Division of Cardiology, Department of Internal Medicine, National Taiwan University Hospital, Hsin-Chu Branch, Hsinchu, Taiwan; 5 Department of Internal Medicine, National Taiwan University College of Medicine, Taipei, Taiwan; 6 Department of Laboratory Medicine, National Taiwan University College of Medicine, Taipei, Taiwan; 7 Cardiovascular Center, National Taiwan University Hospital, Taipei, Taiwan; 8 Telehealth Center, National Taiwan University Hospital, Taipei, Taiwan; 9 Department of Emergency Medicine, National Taiwan University Hospital, Taipei, Taiwan

## Abstract

**Importance:**

Controversy exists regarding whether statin therapy has benefits for patients with kidney failure, and the consequences of statin therapy for patients with kidney failure and concomitant peripheral artery disease (PAD) are particularly uncertain.

**Objective:**

To evaluate the association of statin therapy with cardiovascular (CV) and limb outcomes among patients with kidney failure and concomitant PAD and dyslipidemia who are receiving long-term maintenance dialysis.

**Design, Setting, and Participants:**

This retrospective cohort study used data from the Taiwan National Health Insurance Research Database. A total of 20 731 patients with kidney failure receiving long-term maintenance dialysis who were diagnosed with PAD and dyslipidemia between January 1, 2001, and December 31, 2013, were identified, and 10 767 patients met study criteria. Data were analyzed from June 8, 2021, to June 2, 2022.

**Main Outcomes and Measures:**

Primary outcomes were all-cause death and the composite of endovascular therapy (EVT) and amputation. Other outcomes of interest included CV events (CV death, acute myocardial infarction, ischemic stroke, and hospitalization for heart failure), major adverse limb events (new-onset claudication, new-onset critical limb ischemia, EVT, and nontraumatic amputation), and all-cause readmission. All outcomes were examined at 1 year and 3 years of follow-up. To minimize selection bias, propensity score matching on a 1:1 ratio was performed among patients receiving statin therapy (statin group) and patients not receiving statin therapy (nonstatin group). A defined daily dose (DDD) approach was used to evaluate whether the association of statin therapy with the risk of primary outcomes was dose dependent.

**Results:**

Among 20 731 patients with kidney failure and concomitant PAD and dyslipidemia receiving long-term maintenance dialysis, 10 767 patients (5593 women [51.9%]; mean [SD] age, 68.5 [11.5] years; all of Taiwanese ethnicity) met the predetermined study criteria; of those, 3597 patients were receiving statin therapy, and 7170 were not. A total of 6470 patients (mean [SD] age, 66.4 [11.3] years; 3359 women [51.9%]) were included in the 1:1 propensity score–matched cohort, with 3235 patients in each group (statin and nonstatin). The incidence and risk of CV and all-cause death were significantly lower in the statin group vs the nonstatin group at 3 years of follow-up (CV death: 611 patients [18.9%] vs 685 patients [21.2%]; hazard ratio [HR], 0.86 [95% CI, 0.77-0.96]; *P* = .008; all-cause death: 1078 patients [33.3%] vs 1138 patients [35.2%]; HR, 0.92 [95% CI, 0.84-0.996]; *P* = .04). Statin use was also associated with a significantly lower incidence and risk of the composite adverse limb outcome of EVT and amputation at 3 years of follow-up (314 patients [9.7%] vs 361 patients [11.2%]; subdistribution HR, 0.85 [95% CI, 0.73-0.99]; *P* = .04). Results of subgroup analyses were consistent with those of the primary analysis across all subgroup variables. In the adjusted dose-response analysis, the risk reduction associated with statin use increased in a dose-dependent manner for both all-cause death (HR: 0.95 for DDD <0.50, 0.92 for DDD 0.50-0.99, 0.85 for DDD 1.00-1.49, and 0.79 for DDD ≥1.50; *P* = .002 for trend) and the composite outcome of EVT and amputation (subdistribution HR: 0.79 for DDD <0.50, 0.78 for DDD 0.50-0.99, 0.82 for DDD 1.00-1.49, and 0.58 for DDD ≥1.50; *P* = .002 for trend) compared with no statin therapy; however, not all findings in the DDD analysis were statistically significant.

**Conclusions and Relevance:**

In this cohort study, statin therapy was associated with reductions in the risk of all-cause death, CV death, and the composite adverse limb outcome of EVT and amputation. These findings suggest that statin therapy may have protective CV and limb benefits for patients with kidney failure and concomitant PAD who are receiving long-term maintenance dialysis.

## Key Points

**Question:**

Is statin therapy associated with cardiovascular and limb outcomes among patients with kidney failure and concomitant peripheral artery disease (PAD) and dyslipidemia who are receiving long-term maintenance dialysis?

**Findings:**

In this cohort study involving 20 731 patients with kidney failure and concomitant PAD and dyslipidemia, analysis of a propensity score–matched cohort revealed that the rate of all-cause death was 33.3% among patients who received statin therapy and 35.2% among patients who did not, and the rate of the composite adverse limb outcome of endovascular therapy and amputation was 9.7% among patients who received statin therapy and 11.2% among patients who did not. Both differences were statistically significant.

**Meaning:**

This study’s findings suggest that statin therapy may have protective cardiovascular and limb benefits for patients with kidney failure and concomitant PAD who are receiving long-term maintenance dialysis.

## Teaser

This cohort study assesses the association of statin therapy with cardiovascular and limb outcomes among patients with kidney failure and concomitant peripheral artery disease and dyslipidemia who were receiving long-term maintenance dialysis.

## Introduction

Peripheral artery disease (PAD) has been increasingly prevalent, with more than 200 million people estimated to have PAD worldwide.^1,2^ This disease has been associated with substantially higher risks of all-cause and cardiovascular (CV) death^3^ and is more common among patients with kidney failure than the general population.^4,5^ Patients with PAD who are receiving dialysis are also at higher risk of experiencing major adverse limb events (including amputation and hospitalization for critical limb ischemia [CLI]) and death.^6,7,8,9^

Statin therapy is an important component of treatment for CV disease (CVD) and has been associated with decreases in the risk of all-cause death, CV death, and myocardial infarction (MI).^10^ Statin medications are also recommended for use in patients with PAD^11,12^ and have been associated with lower rates of worsening symptoms, peripheral revascularization, and ischemic amputation.^13,14^ The use of high-intensity statin therapy has been associated with substantial reductions in the incidence of lower limb amputation and death compared with low- to moderate-intensity statin therapy.^15^

Despite many established benefits of statin therapy for the treatment of CVD,^16,17,18^ the use of statin therapy in patients with kidney failure remains controversial. Several previous clinical trials^16,17,18^ did not demonstrate significant benefits associated with statin use in patients receiving dialysis. However, the proportion of patients with PAD included in these clinical trials varied substantially, ranging from 15.3% in the AURORA (A Study to Evaluate the Use of Rosuvastatin in Subjects on Regular Haemodialysis: an Assessment of Survival and Cardiovascular Events) clinical trial^16^ to 45.7% in the 4D (Deutsche Diabetes Dialysis) study.^18^ Because of the heterogenous CV risk backgrounds of the study populations in each clinical trial, forming conclusions regarding whether statin therapy is beneficial for patients with kidney failure at high risk of atherosclerotic disease has been difficult. In addition, adverse limb events were not prespecified end points in any published clinical trials.^16,17,18^ It is largely unknown whether limb-protective actions of statin therapy exist and persist in patients with both kidney failure and PAD. In this cohort study, we aimed to conduct an evaluation in a clinical setting of the association of statin therapy with CV and limb outcomes among patients with kidney failure and concomitant PAD and dyslipidemia who were receiving long-term maintenance dialysis.

## Methods

### Data Source

This cohort study collected data from the Taiwan National Health Insurance Research Database (NHIRD)^19,20^ and the Registry for Catastrophic Illness Patient Database.^21^ More than 99.8% of the approximately 23.7 million people currently living in Taiwan are enrolled in the National Health Insurance (NHI) program^21^ because participation is mandatory. The NHI program is a single-payer system that was established in March 1995 to provide affordable high-quality health care. Data on all individuals covered by the NHI program between 1995 to 2013 are available through application to the NHIRD. Data obtained from the NHIRD are deidentified; therefore, this study was deemed exempt from obtaining informed consent, and a full review of the study protocol was waived by the ethics institutional review board of Taiwan University Hospital. The validity of the data contained in the NHIRD has been confirmed by previous studies.^19,20,21^ This study followed the Strengthening the Reporting of Observational Studies in Epidemiology (STROBE) reporting guideline for cohort studies.

Individuals in Taiwan who have major diseases, such as kidney failure necessitating long-term maintenance dialysis, qualify to apply for catastrophic illness certificates to support long-term medical expenditures. Possession of a catastrophic illness certificate exempts the holder from paying insurance premiums and copayments; therefore, all catastrophic illness certificate applications require supporting laboratory data or histopathological reports and are closely reviewed by the Taiwan Bureau of the NHI program. Individuals with kidney failure who are granted a catastrophic illness certificate are automatically included in the Registry for Catastrophic Illness Patient Database, through which the date of initiation of maintenance dialysis may be extracted.

### Study Cohort

All patients with kidney failure who were receiving long-term maintenance dialysis and diagnosed with PAD and dyslipidemia between January 1, 2001, and December 31, 2013 (N = 20 731), were identified using *International Classification of Diseases, Ninth Revision, Clinical Modification* (*ICD-9-CM*) diagnostic codes (eTable 1 in the Supplement). Data were analyzed from June 8, 2021, to June 2, 2022. The date of kidney failure diagnosis was defined as the index date. Exclusion criteria included (1) missing demographic data (n = 1), (2) age younger than 20 years (n = 20), (3) previous diagnosis of dyslipidemia (n = 8300), (4) previous receipt of a kidney transplant (n = 5), and (5) follow-up for fewer than 90 days (n = 1638). The diagnosis of dyslipidemia was identified through a combination of the *ICD-9-CM* diagnostic code for dyslipidemia and the use of lipid-lowering agents, with high accuracy of diagnosis reported by a previous study.^22^ Patients who were followed up for fewer than 90 days were excluded because of the markedly high short-term mortality rates after initiation of dialysis reported in a previous study of NHIRD data.^23^

**Figure 1.**
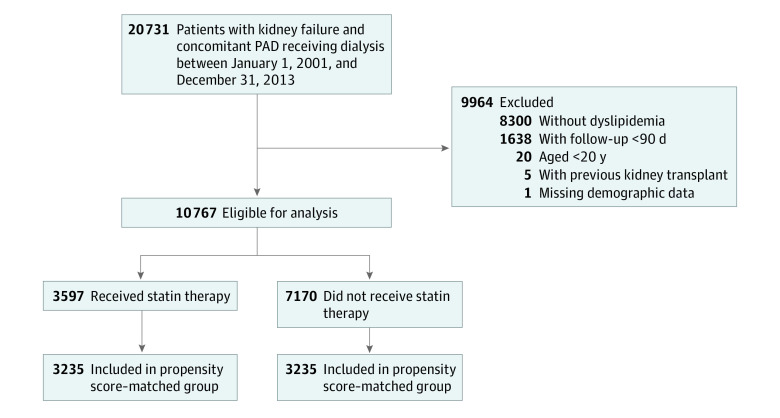
Patient Selection Flowchart PAD indicates peripheral artery disease.

The outcomes of interest in our study were events that occurred during midterm follow-up; therefore, we excluded patients who died within 90 days of initiation of dialysis. Patients were then categorized into 2 groups according to whether they received statin therapy (statin group) or did not receive statin therapy (nonstatin group) (Figure 1). Statin therapy was defined as any prescription for a statin medication filled in the 6 months before the index date as identified in outpatient claims data or long-term medication refill data. In addition, the type of statin medication used was extracted; rosuvastatin, 20 mg to 40 mg, and atorvastatin, 40 mg to 80 mg, were classified as having high potency, and all other statin medications were classified as having low to moderate potency.

### Covariates

The covariates in this study included initial dialysis type, age, sex, comorbid conditions, Charlson Comorbidity Index score, history of events, presence of lower extremity arterial disease (LEAD), and receipt of 21 types of long-term medications. The initial dialysis type specified whether the patient was receiving hemodialysis or peritoneal dialysis when long-term dialysis was initiated. Patients were categorized into 3 age groups: 20 to 64 years, 65 to 74 years, and 75 years and older. Comorbid conditions included diabetes, hypertension, ischemic heart disease, chronic obstructive pulmonary disease, atrial fibrillation, and abnormal liver function. History of events include any previous hospitalization for heart failure (HF), ischemic stroke, systemic embolism, hemorrhagic stroke, and MI, which could be followed up starting in 1995 based on the discharge diagnoses associated with hospital admissions. History of LEAD was defined as any previous documented incidence of claudication or CLI, history of EVT, or amputation. Comorbidities, the presence of LEAD, and Charlson Comorbidity Index scores were included when at least 2 outpatient diagnoses or any single discharge diagnosis was identified for the patient in the previous year. Long-term medications were extracted from prescription records for the previous 6 months. Medications included antidiabetic, anticoagulant, antihypertensive, and antiplatelet agents; insulin; kidney failure–related drugs; and other drugs.

### Outcomes

Primary outcomes were all-cause death and the composite of EVT and amputation. Other outcomes of interest included CV events (acute MI, CV death, hospitalization for HF, ischemic stroke, and the composite of CV death, ischemic stroke, and acute MI), major adverse limb events (EVT for LEAD, new-onset claudication, new-onset CLI, and nontraumatic amputation), and all-cause readmission. Cardiovascular death was defined according to the CV and stroke end point definitions for clinical trials developed by the Standardized Data Collection for Cardiovascular Trials Initiative and the US Food and Drug Administration.^24^ Dates and causes of death were determined using the Registry for Beneficiaries, which is a subdatabase of the NHIRD.^19,20,21^ Hospitalization for acute MI, HF, and ischemic stroke were identified using the principal discharge diagnosis. New-onset CLI was defined as persistent limb, foot, or digit pain when at rest or threatened tissue loss due to ischemia (ie, Fontaine stage III or IV ischemia^25^). New-onset claudication and new-onset CLI required at least 2 outpatient diagnoses or any single inpatient diagnosis. Amputation and EVT were identified using NHI reimbursement codes and inpatient claims data. Most of the *ICD-9-CM* diagnostic codes used in this study (eTable 1 in the Supplement) have been previously validated in NHIRD studies.^21,22,26,27,28,29^ All outcomes were examined at 1 year and 3 years of follow-up.

### Statistical Analysis

To mitigate possible selection bias, propensity score matching was used. The propensity score, defined as the conditional probability of the baseline covariates (shown in Table 1), was calculated using a multivariable logistic regression model in which regression was performed using all covariates in Table 1 (with the follow-up year replaced by the index date) from both study groups (with 1 indicating statin group and 0 indicating nonstatin group) without considering interaction effects among covariates. The matching was processed using a greedy nearest neighbor algorithm with a caliper of 0.20 times the SD of the logit of the propensity score. A random matching order was used, and replacements were not allowed. Patients in the statin and nonstatin groups were matched on a 1:1 ratio. The quality of matching was assessed by the absolute value of the standardized difference between groups after propensity score matching, in which a value of less than 0.10 was considered negligible.

**Table 1.** Baseline Patient Characteristics

Characteristic	Before propensity score matching			After propensity score matching		
	Patients, No. (%)		Standardized difference	Patients, No. (%)		Standardized difference
	Statin group (n = 3597)	Nonstatin group (n = 7170)		Statin group (n = 3235)	Nonstatin group (n = 3235)	
Sex						
Male	1698 (47.2)	3476 (48.5)	−0.03	1546 (47.8)	1565 (48.4)	−0.01
Female	1899 (52.8)	3694 (51.5)	0.03	1689 (52.2)	1670 (51.6)	0.01
Age, mean (SD), y	65.8 (11.4)	68.5 (11.5)	−0.24	66.1 (11.5)	66.6 (11.2)	−0.04
Age group, y						
20-64	1654 (46.0)	2557 (35.7)	0.21	1426 (44.1)	1403 (43.4)	0.01
65-74	1150 (32.0)	2338 (32.6)	−0.01	1059 (32.7)	1075 (33.2)	−0.01
≥75	793 (22.0)	2275 (31.7)	−0.22	750 (23.2)	757 (23.4)	−0.01
Follow-up, mean (SD), y	3.0 (2.4)	2.9 (2.5)	0.03	3.0 (2.5)	2.9 (2.5)	0.03
Initial dialysis type						
Hemodialysis	3230 (89.8)	6650 (92.7)	−0.10	2921 (90.3)	2917 (90.2)	<0.01
Peritoneal dialysis	367 (10.2)	520 (7.3)	0.10	314 (9.7)	318 (9.8)	<0.01
Comorbid conditions						
Diabetes	3052 (84.8)	5555 (77.5)	0.19	2702 (83.5)	2722 (84.1)	−0.02
Hypertension	3406 (94.7)	6601 (92.1)	0.11	3055 (94.4)	3068 (94.8)	−0.02
Ischemic heart disease	1733 (48.2)	3040 (42.4)	0.12	1525 (47.1)	1516 (46.9)	0.01
COPD	288 (8.0)	764 (10.7)	−0.09	275 (8.5)	283 (8.7)	−0.01
Atrial fibrillation	140 (3.9)	350 (4.9)	−0.05	134 (4.1)	127 (3.9)	0.01
Abnormal liver function	307 (8.5)	711 (9.9)	−0.05	279 (8.6)	291 (9.0)	−0.01
Charlson Comorbidity Index score, mean (SD)	5.84 (1.79)	5.75 (2.00)	0.05	5.84 (1.82)	5.85 (1.84)	<0.01
History of events						
Hospitalization for HF	1405 (39.1)	2832 (39.5)	−0.01	1281 (39.6)	1258 (38.9)	0.01
Ischemic stroke	1028 (28.6)	2173 (30.3)	−0.04	937 (29.0)	948 (29.3)	−0.01
Systemic embolism	823 (22.9)	1596 (22.3)	0.01	731 (22.6)	714 (22.1)	0.01
Hemorrhage stroke	88 (2.4)	206 (2.9)	−0.03	81 (2.5)	79 (2.4)	<0.01
MI	594 (16.5)	929 (13.0)	0.10	501 (15.5)	513 (15.9)	−0.01
History of lower extremity atrial disease						
Claudication	130 (3.6)	236 (3.3)	0.02	113 (3.5)	120 (3.7)	−0.01
CLI	633 (17.6)	1255 (17.5)	<0.01	574 (17.7)	561 (17.3)	0.01
History of EVT	261 (7.3)	366 (5.1)	0.09	212 (6.6)	208 (6.4)	0.01
Amputation	388 (10.8)	794 (11.1)	−0.01	352 (10.9)	331 (10.2)	0.02
Any LEAD	1013 (28.2)	1881 (26.2)	0.04	899 (27.8)	872 (27.0)	0.02
Medications						
Antiplatelet	1968 (54.7)	2846 (39.7)	0.30	1688 (52.2)	1659 (51.3)	0.02
Cilostazol	490 (13.6)	717 (10.0)	0.11	416 (12.9)	414 (12.8)	<0.01
Oral anticoagulant	143 (4.0)	260 (3.6)	0.02	132 (4.1)	135 (4.2)	<0.01
ACE inhibitor or ARB	2129 (59.2)	3427 (47.8)	0.23	1860 (57.5)	1841 (56.9)	0.01
β-Blocker	2256 (62.7)	3604 (50.3)	0.25	1977 (61.1)	1965 (60.7)	0.01
DCCB	2842 (79.0)	4899 (68.3)	0.24	2514 (77.7)	2518 (77.8)	<0.01
Loop diuretic	2634 (73.2)	4320 (60.3)	0.28	2316 (71.6)	2321 (71.7)	<0.01
Spironolactone	141 (3.9)	201 (2.8)	0.06	123 (3.8)	120 (3.7)	<0.01
Fibrate	227 (6.3)	562 (7.8)	−0.06	219 (6.8)	224 (6.9)	−0.01
OHA	1794 (49.9)	2856 (39.8)	0.20	1556 (48.1)	1554 (48.0)	<0.01
Insulin	1661 (46.2)	2384 (33.2)	0.27	1410 (43.6)	1397 (43.2)	0.01
Vitamin D	267 (7.4)	464 (6.5)	0.04	234 (7.2)	254 (7.9)	−0.02
Iron supplement	583 (16.2)	943 (13.2)	0.09	506 (15.6)	515 (15.9)	−0.01
Pentoxifylline	706 (19.6)	992 (13.8)	0.16	580 (17.9)	589 (18.2)	−0.01
Sodium bicarbonate	327 (9.1)	582 (8.1)	0.03	291 (9.0)	297 (9.2)	−0.01
Calcium	1090 (30.3)	1856 (25.9)	0.10	966 (29.9)	969 (30.0)	<0.01
Steroid	300 (8.3)	624 (8.7)	−0.01	275 (8.5)	278 (8.6)	<0.01
Immunotherapy	54 (1.5)	86 (1.2)	0.03	47 (1.5)	46 (1.4)	<0.01
Proton pump inhibitor	735 (20.4)	1472 (20.5)	<0.01	673 (20.8)	688 (21.3)	−0.01
NSAID (including COX-2)	581 (16.2)	1243 (17.3)	−0.03	529 (16.4)	537 (16.6)	−0.01
NDCCB	389 (10.8)	634 (8.8)	0.07	333 (10.3)	328 (10.1)	0.01

Abbreviations: ACE, angiotensin-converting enzyme; ARB, angiotensin receptor blocker; CLI, critical limb ischemia; COPD, chronic obstructive pulmonary disease; COX-2, cyclooxygenase-2; DCCB, dihydropyridine calcium channel blocker; EVT, endovascular therapy; HF, heart failure; LEAD, lower extremity arterial disease; MI, myocardial infarction; NDCCB, nondihydropyridine calcium channel blocker; NSAID, nonsteroidal anti-inflammatory drug; OHA, oral hypoglycemic agent.

The risks of fatal outcomes (ie, all-cause death, CV death, and the composite outcome of CV death, ischemic stroke, and acute MI) were compared between the 2 groups using a Cox proportional hazards model and reported as hazard ratios (HRs). The risk of nonfatal outcomes (ie, acute MI, all-cause readmission, claudication, CLI, EVT, hospitalization for HF, ischemic stroke, nontraumatic amputation, and the composite outcome of EVT and amputation) were compared between groups using the Fine and Gray subdistribution hazards model, which considered all-cause death as a competing risk, and were reported as subdistribution HRs (sHRs). The study group (statin vs nonstatin) was the only explanatory variable considered in these survival analyses. Within-pair clustering of outcomes after propensity score matching was accounted for using a robust SE. We performed a sensitivity analysis including patients who were followed up for fewer than 90 days; both propensity score–matched and outcome analyses were conducted.

Subgroup analyses were conducted to evaluate the consistency of the observed associations between treatment and specified outcomes across different levels of subgroup variables. Outcomes of interest included all-cause death and the composite of EVT and amputation. The prespecified subgroup variables of interest included initial type of dialysis (hemodialysis vs peritoneal), patient age (categorized into 3 strata [ages 20-64 years, 65-74 years, or ≥75 years]), patient sex, presence of diabetes, presence of hypertension, presence of ischemic heart disease, Charlson Comorbidity Index score (categorized into 2 groups [score ≤5 or score >5]), history of hospitalization for HF, history of ischemic stroke, history of MI, and presence of preexisting LEAD.

A dose-response analysis of statin potency was conducted using 3 approaches to evaluate the 2 main outcomes (all-cause death and composite of EVT and amputation) at 3 years of follow-up. The dose-response analysis was performed using data from the entire unmatched cohort (n = 10 767). To assess the association between potency and the 2 outcomes, we performed multivariable covariate adjustments for the whole cohort. In the first approach, we compared the incidence and risk of these 2 primary outcomes among patients who received low- to moderate-potency and high-potency statin therapy with the risks among patients who did not receive statin therapy. The linear trend for potency (with 0 indicating nonstatin, 1 indicating low to moderate potency, and 2 indicating high potency) across the risk of outcomes was also assessed. In the second approach, we evaluated associations using defined daily dose (DDD), with patients classified into 5 groups (nonstatin, DDD <0.50, DDD 0.50-0.99, DDD 1.00-1.49, and DDD ≥1.50). In addition, the linear trend for ordinal DDD groups across the risk of outcomes was evaluated. In the third approach, the use of statin medications was treated as a time-varying exposure. The status of statin use was reassessed every 3 months after the index date during the entire 3-year follow-up period. The periods of follow-up for each patient were classified as nonstatin, low to moderate potency, and high potency. The linear trend for time-varying exposure to statin medications across the risk of outcomes was also assessed. All of the baseline characteristics (Table 1) were adjusted in the analysis, in which the follow-up year was replaced by the index date.

The adjusted survival rates for all-cause death and the fitted cumulative incidence functions for the composite outcome of EVT and amputation were calculated based on methods proposed in previous studies.^30,31^ The threshold for statistical significance was 2-tailed *P* < .05. All statistical analyses were performed using SAS software, version 9.4 (SAS Institute Inc).

## Results

### Inclusion of Participants

A total of 20 731 patients who met inclusion criteria were identified between January 1, 2001, and December 31, 2013. After applying the predetermined exclusion criteria, a total of 10 767 patients (5593 women [51.9%] and 5174 men [48.1%]; mean [SD] age, 68.5 [11.5] years; all of Taiwanese ethnicity) were included in the analysis. Of those, 3597 patients were receiving statin therapy, and 7170 patients were not. Before propensity score matching, patients in the statin group were followed up for a mean (SD) of 3.0 (2.4) years, whereas those in the nonstatin group were followed up for a mean (SD) of 2.9 (2.5) years. After 1:1 propensity score matching, 6470 patients (mean [SD] age, 66.4 [11.3] years; 3359 women [51.9%] and 3111 men [48.1%]) were included, with 3235 patients in each group (statin vs nonstatin) (Figure 1).

### Baseline Characteristics of Statin and Nonstatin Groups

The baseline characteristics of the entire unmatched and propensity score–matched cohorts are shown in Table 1. Before propensity score matching, compared with patients in the nonstatin group, those in the statin group were younger (mean [SD], 65.8 [11.4] years vs 68.5 [11.5] years; standardized difference, −0.24), were less likely to be receiving hemodialysis (3230 patients [89.8%] vs 6650 patients [92.7%]; standardized difference, −0.10), and had a higher prevalence of diabetes (3052 patients [84.8%] vs 5555 patients [77.5%]; standardized difference, 0.19), hypertension (3406 patients [94.7%] vs 6601 patients [92.1%]; standardized difference, 0.11), ischemic heart disease (1733 patients [48.2%] vs 3040 patients [42.4%]), previous MI (594 patients [16.5%] vs 929 patients [13.0%]; standardized difference, 0.10), and prescriptions for antiplatelet agents (1968 patients [54.7%] vs 2846 patients [39.7%]; standardized difference, 0.30), cilostazol (490 patients [13.6%] vs 717 patients [10.0%]; standardized difference, 0.11), antihypertensive agents (eg, angiotensin-converting enzyme inhibitor or angiotensin receptor blocker: 2129 patients [59.2%] vs 3427 patients [47.8%]; standardized difference, 0.23), β-blockers (2256 patients [62.7%] vs 3604 patients [50.3%]; standardized difference, 0.25), diuretic agents (eg, loop diuretic: 2634 patients [73.2%] vs 4320 patients [60.3%]; standardized difference, 0.28), oral hypoglycemic agents (1794 patients [49.9%] vs 2856 patients [39.8%]; standardized difference, 0.20), and insulin (1661 patients [46.2%] vs 2384 patients [33.2%]; standardized difference, 0.27) at baseline.

Before propensity score matching, the incidence of LEAD (1013 patients [28.2%] vs 1881 patients [26.2%]; standardized difference, 0.04) and related comorbidities, including claudication (130 patients [3.6%] vs 236 patients [3.3%]; standardized difference, 0.03), CLI (633 patients [17.6%] vs 1255 patients [17.5%]; standardized difference, <0.01), history of EVT (261 patients [7.3%] vs 366 patients [5.1%]; standardized difference, 0.09), and amputation (388 patients [10.8%] vs 794 patients [11.1%]; standardized difference, −0.01), were present in similar proportions in the statin group vs the nonstatin group. After propensity score matching, all baseline characteristics were well balanced between the groups, with absolute standardized differences lower than 0.10 for all examined variables (Table 1).

### Cardiovascular and Other Outcomes

At 1 year of follow-up, patients in the statin group had a significantly lower incidence and risk of CV death compared with those in the nonstatin group (250 patients [7.7%] vs 295 patients [9.1%]; HR, 0.83; 95% CI; 0.70-0.98; *P* = .03) (Table 2). No differences between the statin and nonstatin groups were noted for the occurrence of ischemic stroke (103 patients [3.2%] vs 92 patients [2.8%]; sHR, 1.11; 95% CI, 0.84-1.47; *P* = .47); acute MI (107 patients [3.3%] vs 98 patients [3.0%]; sHR, 1.08; 95% CI, 0.82-1.42; *P* = .58); the composite outcome of CV death, ischemic stroke, and acute MI (420 patients [13.0%] vs 441 patients [13.6%]; HR, 0.93; 95% CI, 0.82-1.06; *P* = .30); or hospitalization for HF (155 patients [4.8%] vs 126 patients [3.9%]; sHR, 1.23; 95% CI, 0.97-1.56; *P* = .10). Rates of all-cause death (430 patients [13.3%] vs 468 patients [14.5%]; HR, 0.89; 95% CI, 0.78-1.02; *P* = .09) and all-cause readmission (2107 patients [65.1%] vs 2090 patients [64.6%]; sHR, 0.99; 95% CI, 0.93-1.05; *P* = .80) were also similar between groups at 1 year of follow-up.

**Table 2.** Major Adverse Cardiovascular and Limb Outcomes in Propensity Score–Matched Cohort

Outcome	Patients, No. (%)		HR or sHR for statin use (95% CI)^a^	*P* value
	Statin group (n = 3235)	Nonstatin group (n = 3235)		
At 1-y of follow-up				
CV events				
CV death	250 (7.7)	295 (9.1)	0.83 (0.70-0.98)	.03
All-cause death	430 (13.3)	468 (14.5)	0.89 (0.78-1.02)	.09
Ischemic stroke	103 (3.2)	92 (2.8)	1.11 (0.84-1.47)	.47
Acute MI	107 (3.3)	98 (3.0)	1.08 (0.82-1.42)	.58
Composite of CV death, ischemic stroke, and acute MI	420 (13.0)	441 (13.6)	0.93 (0.82-1.06)	.30
Hospitalization for HF	155 (4.8)	126 (3.9)	1.23 (0.97-1.56)	.10
All-cause readmission	2107 (65.1)	2090 (64.6)	0.99 (0.93-1.05)	.80
Major adverse limb events				
New-onset claudication	30 (0.9)	38 (1.2)	0.78 (0.49-1.26)	.31
New-onset CLI	95 (2.9)	113 (3.5)	0.83 (0.63-1.09)	.18
EVT	91 (2.8)	110 (3.4)	0.82 (0.62-1.08)	.16
Nontraumatic amputation	98 (3.0)	131 (4.0)	0.74 (0.57-0.96)	.02
Composite of EVT and amputation	159 (4.9)	194 (6.0)	0.81 (0.65-0.99)	.04
At 3-y of follow-up				
CV events				
CV death	611 (18.9)	685 (21.2)	0.86 (0.77-0.96)	.008
All-cause death	1078 (33.3)	1138 (35.2)	0.92 (0.84-0.996)	.04
Ischemic stroke	255 (7.9)	226 (7.0)	1.13 (0.94-1.35)	.20
Acute MI	229 (7.1)	194 (6.0)	1.18 (0.97-1.42)	.10
Composite of CV death, ischemic stroke, and acute MI	921 (28.5)	967 (29.9)	0.93 (0.85-1.02)	.12
Hospitalization for HF	244 (7.5)	221 (6.8)	1.10 (0.92-1.32)	.30
All-cause readmission	2648 (81.9)	2617 (80.9)	1.00 (0.95-1.06)	.91
Major adverse limb events				
New-onset claudication	50 (1.5)	63 (1.9)	0.79 (0.54-1.14)	.20
New-onset CLI	212 (6.6)	215 (6.6)	0.98 (0.81-1.18)	.80
EVT	180 (5.6)	211 (6.5)	0.84 (0.69-1.03)	.09
Nontraumatic amputation	215 (6.6)	248 (7.7)	0.85 (0.71-1.02)	.08
Composite of EVT and amputation	314 (9.7)	361 (11.2)	0.85 (0.73-0.99)	.04

Abbreviations: CLI, critical limb ischemia; CV, cardiovascular; EVT, endovascular therapy; HF, heart failure; MI, myocardial infarction.

^a^ Hazard ratios (HRs) were used for fatal outcomes (all-cause death, CV death, and the composite of CV death, ischemic stroke, and acute MI), and subdistribution HRs (sHRs) were used for nonfatal outcomes (acute MI, all-cause readmission, claudication, CLI, EVT, hospitalization for HF, ischemic stroke, nontraumatic amputation, and the composite outcome of EVT and amputation).

**Figure 2.**
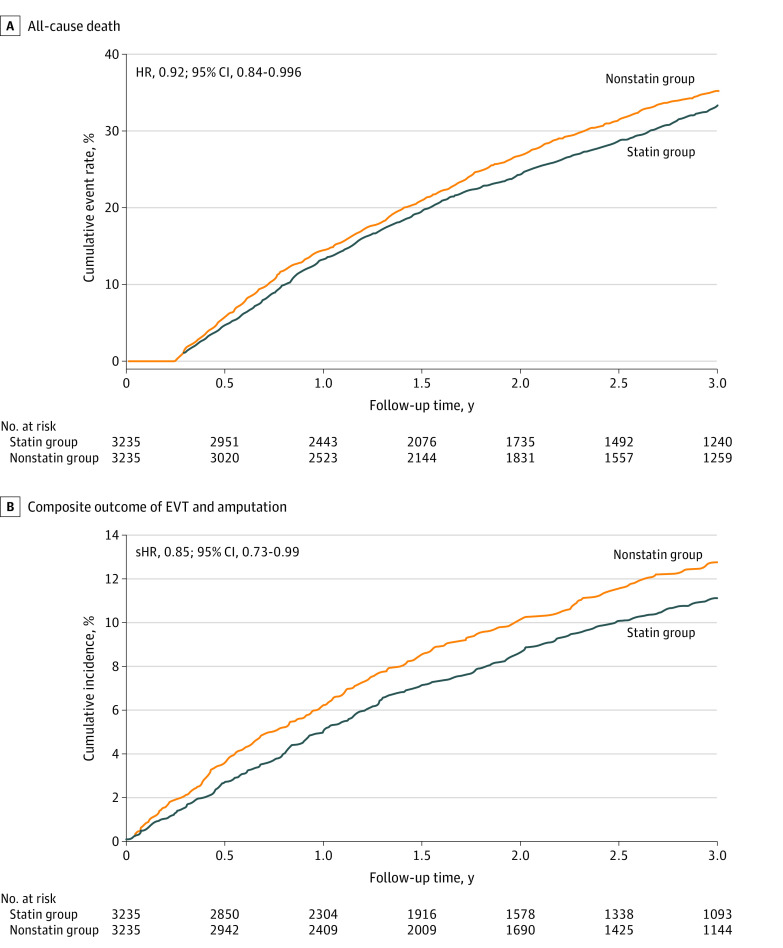
Cumulative Event Rate of All-Cause Death and Cumulative Incidence of Composite EVT and Amputation Analysis of propensity score–matched groups. EVT indicates endovascular therapy; and HR, hazard ratio; and sHR, subdistribution HR.

The incidence and risk of CV death remained significantly lower in the statin group vs the nonstatin group after 3 years of follow-up (611 patients [18.9%] vs 685 patients [21.2%]; HR, 0.86; 95% CI, 0.77-0.96; *P* = .008). The risk of all-cause death was also significantly lower in the statin group compared with the nonstatin group at 3 years (1078 patients [33.3%] vs 1138 patients [35.2%]; HR, 0.92; 95% CI, 0.84-0.996; *P* = .04) (Figure 2A). The incidence and risk of other outcomes, including ischemic stroke (255 patients [7.9%] vs 226 patients [7.0%]; sHR, 1.13; 95% CI, 0.94-1.35; *P* = .20); acute MI (229 patients [7.1%] vs 194 patients [6.0%]; sHR, 1.18; 95% CI, 0.97-1.42; *P* = .10); the composite outcome of CV death, ischemic stroke, and acute MI (921 patients [28.5%] vs 967 patients [29.9%]; HR, 0.93; 95% CI, 0.85-1.02; *P* = .12); hospitalization for HF (244 patients [7.5%] vs 221 patients [6.8%]; sHR, 1.10; 95% CI, 0.92-1.32; *P* = .30); and all-cause readmission (2648 patients [81.9%] vs 2617 patients [80.9%]; sHR, 1.00; 95% CI, 0.95-1.06; *P* = .91) were similar between the statin and nonstatin groups at 3 years of follow-up.

### Major Adverse Limb Events

The use of statin medications was associated with significant reductions in the incidence and risk of nontraumatic amputation (98 patients [3.0%] vs 131 patients [4.0%]; sHR, 0.74; 95% CI, 0.57-0.96; *P* = .02) and the composite outcome of EVT and amputation (159 patients [4.9%] vs 194 patients [6.0%]; sHR, 0.81; 95% CI, 0.65-0.99; *P* = .04) compared with the nonuse of statin medications at 1 year of follow-up (Table 2). No differences in the incidence and risk of new-onset claudication (30 patients [0.9%] vs 38 patients [1.2%]; sHR, 0.78; 95% CI, 0.49-1.26; *P* = .31), new-onset CLI (95 patients [2.9%] vs 113 patients [3.5%]; sHR, 0.83; 95% CI, 0.63-1.09; *P* = .18), or EVT (91 patients [2.8%] vs 110 patients [3.4%]; sHR, 0.82; 95% CI, 0.62-1.08; *P* = .16) were observed between the statin and nonstatin groups.

After 3 years of follow-up, the statin group had a lower incidence and risk of the composite outcome of EVT and amputation compared with the nonstatin group (314 patients [9.7%] vs 361 patients [11.2%]; sHR, 0.85; 95% CI, 0.73-0.99; *P* = .04) (Figure 2B). The incidence and risk of new-onset claudication (50 patients [1.5%] vs 63 patients [1.9%]; sHR, 0.79; 95% CI, 0.54-1.14; *P* = .20), new-onset CLI (212 patients [6.6%] vs 215 patients [6.6%]; sHR, 0.98; 95% CI, 0.81-1.18; *P* = .80), EVT (180 patients [5.6%] vs 211 patients [6.5%]; sHR, 0.84; 95% CI, 0.69-1.03; *P* = .09), and nontraumatic amputation (215 patients [6.6%] vs 248 patients [7.7%]; sHR, 0.85; 95% CI, 0.71-1.02; *P* = .08) were not significantly different between the statin and nonstatin groups at 3 years of follow-up.

### Sensitivity and Subgroup Analyses

Patients who were followed up for fewer than 90 days were excluded from the primary analysis. Because this exclusion may have produced selection bias, a sensitivity analysis was performed in which both the propensity score–matched analysis and the outcome analysis were repeated without excluding patients who were followed up for fewer than 90 days (3642 patients in each group). The results of the sensitivity analysis were consistent with those of the primary analysis (eg, all-cause death at 3 years of follow-up: 1358 patients [37.3%] in the statin group vs 1432 patients [39.3%] in the nonstatin group; HR, 0.92 [95% CI, 0.85-0.99]; *P* = .02; composite outcome of EVT and amputation at 3 years of follow-up: 312 patients [8.6%] in the statin group vs 392 patients [10.8%] in the nonstatin group; sHR, 0.78 [95% CI, 0.67-0.90]; *P* = .001) (eTable 2 in the Supplement).

In the subgroup analyses of both all-cause death and the composite outcome of EVT and amputation, the protective benefits of statin therapy remained consistent across all levels of subgroup variables, as revealed by significances for interactions greater than or equal to .05 for all variables (eg, presence of diabetes: *P* = .78 for interaction; presence of ischemic heart disease: *P* = .13 for interaction; history of HF: *P* = .98 for interaction; history of ischemic stroke: *P* = .06 for interaction; previous lower extremity arterial disease: *P* = .36 for interaction) (eFigure 1 in the Supplement).

### Dose-Response Analysis

Three different approaches were used to evaluate the potential dose-response association between statin potency and outcomes. The first approach, which compared the incidence of primary outcomes in the low- to moderate-potency and high-potency statin groups vs the nonstatin group, revealed that the event rates for all-cause death were 2865 of 7170 patients (40.0%) in the nonstatin group, 1020 of 3009 patients (33.9%) in the low- to moderate-potency statin group, and 151 of 588 patients (25.7%) in the high-potency statin group. The event rates for the composite outcome of EVT and amputation were 729 of 7170 patients (10.2%) in the nonstatin group, 298 of 3009 patients (9.9%) in the low- to moderate-potency statin group, and 55 of 588 patients (9.4%) in the high-potency statin group. These results suggested that the risk reduction associated with statin therapy increased with the use of higher-potency drugs for both outcomes (eFigure 2 in the Supplement).

The second approach was a DDD analysis, with the adjusted model revealing that higher statin dose was associated with lower risk of both all-cause death (HR: 0.95 for DDD <0.50, 0.92 for DDD 0.50-0.99, 0.85 for DDD 1.00-1.49, and 0.79 for DDD ≥1.50; *P* = .002 for trend) and the composite outcome of EVT and amputation (sHR: 0.79 for DDD <0.50, 0.78 for DDD 0.50-0.99, 0.82 for DDD 1.00-1.49, and 0.58 for DDD ≥1.50; *P* = .002 for trend) compared with no statin use. However, not all DDD findings were statistically significant (Table 3).

**Table 3.** Dose-Response Analysis of Association Between Statin Potency and Primary Outcomes

Outcome	Patients, No.	Events, No. (%)	Unadjusted analysis		Adjusted analysis^a^	
			HR or sHR (95% CI)^b^	*P* value for trend	HR or sHR (95% CI)^b^	*P* value for trend
**All-cause death, DDD**						
0	7170	2865 (40.0)	1 [Reference]	<.001	1 [Reference]	.002
<0.50	797	268 (33.6)	0.81 (0.71-0.92)		0.95 (0.83-1.08)	
0.50-0.99	1720	572 (33.3)	0.78 (0.71-0.85)		0.92 (0.84-1.01)	
1.00-1.49	946	293 (31.0)	0.74 (0.66-0.83)		0.85 (0.75-0.97)	
≥1.50	134	38 (28.4)	0.66 (0.48-0.91)		0.79 (0.57-1.09)	
**Composite EVT and amputation, DDD**						
0	7170	729 (10.2)	1 [Reference]	.95	1 [Reference]	.002
<0.50	797	71 (8.9)	0.88 (0.69-1.12)		0.79 (0.61-1.01)	
0.50-0.99	1720	169 (9.8)	0.96 (0.81-1.13)		0.78 (0.66-0.94)	
1.00-1.49	946	101 (10.7)	1.07 (0.87-1.32)		0.82 (0.66-1.03)	
≥1.50	134	12 (9.0)	0.89 (0.50-1.57)		0.58 (0.31-1.08)	

Abbreviations: DDD, defined daily dose; EVT, endovascular therapy.

^a^ All of the baseline characteristics listed in Table 1 were adjusted in this analysis, with the follow-up year replaced by the index date.

^b^ Hazard ratios (HRs) were used for fatal outcomes (all-cause death, CV death, and the composite of CV death, ischemic stroke, and acute MI), and subdistribution HRs (sHRs) were used for nonfatal outcomes (acute MI, all-cause readmission, claudication, critical limb ischemia, EVT, hospitalization for heart failure, ischemic stroke, nontraumatic amputation, and the composite outcome of EVT and amputation).

The third approach, which comprised a time-varying analysis of statin potency, yielded results that were consistent with those of the first 2 approaches. However, the linear trend in the adjusted time-varying analysis of the association between statin potency and the risk of the composite outcome of EVT and amputation was not statistically significant (sHR, 0.90 for low- to moderate-potency period vs 0.85 for high-potency period; *P* = .11 for trend) compared with no statin use, which may be explained by the limited periods during which patients were exposed to high-potency statin therapy (eTable 3 in the Supplement).

## Discussion

In this retrospective cohort study, we investigated the association of statin therapy with the incidence and risk of CV and major adverse limb outcomes among patients with kidney failure and concomitant PAD and dyslipidemia who were receiving long-term maintenance dialysis. Our results suggested that statin therapy was protective against CV death and the composite outcome of EVT and amputation, with benefits evident starting at 1 year of follow-up and persisting through 3 years of follow-up. After 3 years of follow-up, statin therapy was also associated with a reduced risk of all-cause death. In the present cohort, the benefits of statin therapy were consistent regardless of dialysis type, patient age or sex, or underlying comorbidities. The protective benefits of statin medications were positively associated with potency, with more substantial reductions in all-cause death and the composite outcome of EVT and amputation observed among patients who received treatment with rosuvastatin or atorvastatin. Among patients with PAD receiving maintenance dialysis, statin therapy may be associated with CV benefits that are worth further investigation.

Controversy exists regarding whether the CV benefits observed with the use of statin therapy among the general population are also present in patients with kidney failure. Data on patients receiving dialysis are scarce. The potential lack of CV benefits associated with statin use among patients with kidney failure was first suggested by the results of the 4D study,^18^ in which treatment with atorvastatin did not reduce the primary composite outcome of CV death, MI, and stroke compared with placebo. In the AURORA study that followed,^16^ treatment with rosuvastatin was also no different than placebo for the primary composite outcome. The SHARP (Study of Heart and Renal Protection) clinical trial^17^ similarly found that the risks of atherosclerotic events among patients receiving dialysis were similar between the simvastatin and ezetimibe group and the control group. In a subsequent meta-analysis^32^ examining the consequences of kidney function for CV benefits associated with statin therapy, the reduction in CV events was found to be nonsignificant, with estimated glomerular filtration rates lower than 30 mL/min/1.73 m^2^. Several explanations have been proposed. Although the risk of CVD is higher among patients with chronic kidney disease, reaching a maximum level among those receiving dialysis, the association between risk of CVD and severity of kidney disease was found to be independent of conventional risk factors such as hyperlipidemia^33^; therefore, lipid-reducing therapies may have limited benefits for the reduction of CV risk in these patients. Furthermore, among patients with kidney failure, CV deaths associated with HF or sudden cardiac death are more common than those associated with atherosclerotic etiologies.^33^ Thus, the use of statin medications to prevent CV events in patients receiving dialysis has been insufficiently supported by the existing literature.^16,17,18^

Despite the negative findings from randomized clinical trials, real-world observational studies have reported benefits of statin therapy for patients with kidney failure. In a retrospective study, Jung et al^34^ found a reduction in the risk of all-cause death associated with statin use among patients who began maintenance dialysis during the study period. Jung et al^34^ also reported that patients who were receiving statin therapy before dialysis but discontinued use before or at initiation of dialysis had worse survival outcomes than those who were never exposed to statin therapy. Similar findings were found in a retrospective study^35^ of patients who were receiving statin therapy before the kidney failure diagnosis; this study compared patients who discontinued statin use after the transition to dialysis with patients who continued statin use, finding that persistent statin use was associated with lower all-cause and CV death. Patients with chronic kidney disease who were prescribed statin medications exhibited features of high atherosclerotic risk; these patients may have benefitted from statin therapy even after initiation of dialysis.^35^ Statin therapy has also been associated with reductions in all-cause death among patients with kidney failure who had established atherosclerotic CVD^36^ and with a reduction in the risk of the composite outcome of MI, stroke, and all-cause death after percutaneous coronary intervention with stenting.^37^ Our study enrolled patients with kidney failure who were diagnosed with dyslipidemia and had established PAD; this population constitutes a group of patients with a substantially high risk of atherosclerotic CV events.^38^ This high baseline risk level likely explains the positive findings for statin therapy in our study compared with the lack of benefits reported by previous randomized clinical trials.^16,17,18^

The benefits of statin therapy for limb protection in patients with PAD have been well established. In a study of data from the REACH (Reduction of Atherothrombosis for Continued Health) registry,^13^ statin use was associated with an 18% reduction in the primary adverse limb outcome, which was a composite of worsening claudication or new CLI episodes, new percutaneous or surgical revascularization, and amputation. Similar results were also reported in other cohort studies,^39,40,41^ and results from meta-analyses have also been consistent.^42,43,44^ Concerns regarding increased vascular calcification with statin use have been raised,^45^ and the association between vascular calcification and CV events remains uncertain. A recent cohort study^46^ reported a higher incidence of PAD among patients receiving regular hemodialysis who were diagnosed with dyslipidemia and found a 1.35-fold increase in the risk of new PAD diagnoses among patients receiving statin therapy. However, this study did not report the occurrence of outcomes such as revascularization, amputation, new-onset claudication, or CLI.^46^ In addition, diagnoses of PAD were based on ankle-brachial index measurements,^46^ which are not consistently correlated with the symptom severity of PAD.^11^ Our study found that statin use was associated with a reduction in the composite outcome of EVT and amputation among patients with PAD who were receiving maintenance dialysis. The association between statin therapy and reductions in adverse outcomes observed in our study was consistent with previous findings in the literature.^13,39,40,41,42,43,44^

Current guidelines recommend the use of high-intensity statin therapy for the treatment of patients with symptomatic PAD,^47^ and the benefits of high-intensity statin therapy have been reported in previous studies.^15,48^ In an observational study^15^ of patients with PAD, both high-intensity and low- to moderate-intensity statin therapies were associated with reductions in the risk of amputation and death compared with no statin therapy. The extent of risk reduction was greatest among patients who received high-intensity formulations, but the results of tests for trend were not reported.^15^ Patients with chronic kidney disease or kidney failure constituted very small proportions of the study populations in previous studies.^15,48^ In the present study, we not only observed greater benefit with regard to all-cause death and the composite outcome of EVT and amputations with the use of high-intensity statin therapy compared with low- to moderate-intensity statin therapy among patients with PAD, we also found that this phenomenon occurred among patients receiving long-term maintenance dialysis.

### Limitations

This study has several limitations. First, details regarding symptoms and disease severity were unavailable in the NHIRD. We sought to overcome this limitation by extracting all diagnostic or procedural codes associated with PAD severity, including the presence of claudication, CLI, previous EVT, and nontraumatic amputation. However, determining whether the benefits of statin therapy vary across patients with different symptom severities requires further prospective investigations.

Second, the behavior of patients could not be assessed using data from the NHIRD database. Prescription of statin medications does not guarantee adherence to treatment. The goal of this study was to evaluate the real-world outcomes of statin use for the prevention of limb and CV adverse events among patients with kidney failure and concomitant PAD and dyslipidemia. Thus, this limitation is inevitable because it is inherent in real-world cohort studies.

Third, a number of associations were tested in this study, with some of them producing small effect sizes despite their statistical significance. The results of the study were generally exploratory rather than confirmatory. Conclusions derived from this study are conservative and require further prospective studies with larger samples for validation.

Fourth, retrospective observational database studies such as ours are likely to be subject to confounding factors that may have implications for results. Propensity score matching was conducted to minimize the consequences of measured confounders, but potential selection bias from unobservable variables could have produced bias in the main analysis. Therefore, additional contributions from prospective randomized studies are needed to provide stronger support for our findings.

## Conclusions

This cohort study found that statin therapy was associated with reductions in the risk of all-cause death, CV death, and adverse limb outcomes, including the composite outcome of EVT and amputation. These findings suggest that statin therapy may have protective CV and limb benefits for patients with kidney failure and concomitant PAD who are receiving long-term maintenance dialysis.

## Notes

Supplement. **eTable 1.** *ICD-9-CM* Diagnostic Codes Used in This Study **eTable 2.** Major Adverse Cardiovascular and Limb Events Among Patients With Peripheral Artery Disease Receiving Dialysis Who Were and Were Not Prescribed Statin Medications in the Propensity Score–Matched Cohort Without Excluding Patients Followed Up for Fewer Than 90 Days **eTable 3.** Sensitivity Analysis Treating Different Statin Potency Levels as Time-Varying Exposures to Assess Association With the Risk of All-Cause Death and the Composite Outcome of Endovascular Therapy and Amputation in the Whole Cohort **eFigure 1.** Subgroup Analysis of the Association of Statin Therapy With the Risk of All-Cause Death and the Composite Outcome of Endovascular Therapy and Amputation in the Propensity Score–Matched Cohort **eFigure 2.** Adjusted One-Minus Survival Rates of All-Cause Death and Fitted Cumulative Incidence Functions of the Composite Outcome of Endovascular Therapy and Amputation According to Different Statin Potency Levels
