# Supplementary material for: Association of Statin Therapy With Major Adverse Cardiovascular and Limb Outcomes in Patients With End-stage Kidney Disease and Peripheral Artery Disease Receiving Maintenance Dialysis
Source: JAMA Netw Open. 2022 Sep 1;5(9):e2229706. doi: 10.1001/jamanetworkopen.2022.29706 (PMC9437764; doi:10.1001/jamanetworkopen.2022.29706)
Supplement: Supplement. — eTable 1. ICD-9-CM Diagnostic Codes Used in This Study eTable 2. Major Adverse Cardiovascular and Limb Events Among Patients With Peripheral Artery Disease Receiving Dialysis Who Were and Were Not Prescribed Statin Medications in the Propensity Score–Matched Cohort Without Excluding Patients Followed Up for Fewer Than 90 Days eTable 3. Sensitivity Analysis Treating Different Statin Potency Levels as Time-Varying Exposures to Assess Association With the Risk of All-Cause Death and the Composite Outcome of Endovascular Therapy and Amputation in the Whole Cohort eFigure 1. Subgroup Analysis of the Association of Statin Therapy With the Risk of All-Cause Death and the Composite Outcome of Endovascular Therapy and Amputation in the Propensity Score–Matched Cohort eFigure 2. Adjusted One-Minus Survival Rates of All-Cause Death and Fitted Cumulative Incidence Functions of the Composite Outcome of Endovascular Therapy and Amputation According to Different Statin Potency Levels [file jamanetwopen-e2229706-s001.pdf]

# Supplementary Online Content

Lo HY, Lin YS, Lin DSH, Lee JK, Chen WJ. Association of statin therapy with major adverse cardiovascular and limb outcomes in patients with end-stage kidney disease and peripheral artery disease receiving maintenance dialysis. *JAMA Netw Open*. 2022;5(9):e2229706. doi:10.1001/jamanetworkopen.2022.29706

**eTable 1.** *ICD-9-CM* Diagnostic Codes Used in This Study

**eTable 2.** Major Adverse Cardiovascular and Limb Events Among Patients With Peripheral Artery Disease Receiving Dialysis Who Were and Were Not Prescribed Statin Medications in the Propensity Score–Matched Cohort Without Excluding Patients Followed Up for Fewer Than 90 Days

**eTable 3.** Sensitivity Analysis Treating Different Statin Potency Levels as Time-Varying Exposures to Assess Association With the Risk of All-Cause Death and the Composite Outcome of Endovascular Therapy and Amputation in the Whole Cohort

**eFigure 1.** Subgroup Analysis of the Association of Statin Therapy With the Risk of All-Cause Death and the Composite Outcome of Endovascular Therapy and Amputation in the Propensity Score–Matched Cohort

**eFigure 2.** Adjusted One-Minus Survival Rates of All-Cause Death and Fitted Cumulative Incidence Functions of the Composite Outcome of Endovascular Therapy and Amputation According to Different Statin Potency Levels

This supplementary material has been provided by the authors to give readers additional information about their work.

© 2022 Lo HY et al. *JAMA Network Open*.

**eTable 1.** *ICD-9-CM* Diagnostic Codes Used in This Study

| Disease | ICD-9-CM diagnostic codes |
| --- | --- |
| Peripheral artery disease | 440.0.xx, 440.2x, 440.3x, 440.8x, 440.9x, 443.xx, 444.0x, 444.22, 444.8x, 447.8x, 447.9x |
| Dyslipidemia | 272.xx |
| Kidney transplant | Using Taiwan NHI reimbursement codes |
| Diabetes mellitus | 250.xx |
| Hypertension | 401.xx-405.xx |
| Ischemic heart disease | 410.xx-414.xx |
| Chronic obstructive pulmonary disease | 491.xx, 492.xx, 496.xx |
| Atrial fibrillation | 427.31 |
| Abnormal liver function | 070.xx, 456.0–456.2, 570.xx, 571.xx, 572.2–572.8, 573.xx, V42.7 |
| Heart failure | 428.xx |
| Ischemic stroke | 433.xx-437.xx |
| Systemic embolism | 444.22, 444.81, 444.21, 362.30, 362.34, 593.81, 444.89, 557.0, 557.9, 557.1, 444.9x |
| Hemorrhage stroke | 430.xx-432.xx |
| Myocardial infarction | 410.xx, 412.xx |
| Claudication | 440.21 |
| Critical limb ischemia | 440.22, 440.23, 440.24 |
| Endovascular therapy | 440.xx, 441.xx, 443.xx, 444.0x, 444.8x, 447.8x, 447.9x, 093.0, 437.3, 444.22, 447.1, 557.1, 557.9, V434 and using Taiwan NHI reimbursement codes |
| Amputation | Using Taiwan NHI reimbursement codes |

ICD-9-CM, International Classification of Diseases, Ninth Revision, Clinical Modification.

© 2022 Lo HY et al. *JAMA Network Open*.

**eTable 2.** Major Adverse Cardiovascular and Limb Events Among Patients With Peripheral Artery Disease Receiving Dialysis Who Were and Were Not Prescribed Statin Medications in the Propensity Score–Matched Cohort Without Excluding Patients Followed Up for Fewer Than 90 Days

| Outcome | Statin-user (*n* = 3,642) | Non-statin user (*n* = 3,642) | HR or SHR for statin user (95% CI) | *P* value |
|---|---|---|---|---|
| At 1-year follow up | | | | |
| Cardiovascular outcomes | | | | |
| Cardiovascular death | 413 (11.3) | 468 (12.9) | 0.87 (0.76–0.99) | 0.03 |
| All-cause mortality | 706 (19.4) | 760 (20.9) | 0.91 (0.83–1.01) | 0.07 |
| Ischemic stroke (IS) | 105 (2.9) | 108 (3.0) | 0.97 (0.74–1.26) | 0.80 |
| Acute myocardial infarction (AMI) | 117 (3.2) | 111 (3.0) | 1.05 (0.81–1.36) | 0.73 |
| Composite of cardiovascular death, IS or AMI | 589 (16.2) | 633 (17.4) | 0.91 (0.82–1.02) | 0.10 |
| Heart failure hospitalization | 161 (4.4) | 122 (3.3) | 1.32 (1.04–1.67) | 0.02 |
| All-cause readmission | 2,262 (62.1) | 2,289 (62.9) | 0.97 (0.91–1.03) | 0.26 |
| Major adverse limb events | | | | |
| Newly-onset claudication | 30 (0.8) | 35 (1.0) | 0.85 (0.53–1.38) | 0.52 |
| Newly-onset critical limb ischemia | 105 (2.9) | 142 (3.9) | 0.73 (0.57–0.94) | 0.01 |
| Endovascular therapy (EVT) | 88 (2.4) | 129 (3.5) | 0.67 (0.51–0.89) | 0.01 |
| Non-traumatic amputation | 100 (2.7) | 161 (4.4) | 0.61 (0.48–0.78) | <0.001 |
| Composite outcome of EVT and amputation | 157 (4.3) | 226 (6.2) | 0.68 (0.56–0.84) | <0.001 |
| At 3-year follow up | | | | |
| Cardiovascular outcomes | | | | |

© 2022 Lo HY et al. *JAMA Network Open*.

| Outcome | Statin-user (*n* = 3,642) | Non-statin user (*n* = 3,642) | HR or SHR for statin user (95% CI) | *P* value |
|---|---|---|---|---|
| Cardiovascular death | 778 (21.4) | 862 (23.7) | 0.87 (0.79–0.96) | 0.006 |
| All-cause mortality | 1,358 (37.3) | 1,432 (39.3) | 0.92 (0.85–0.99) | 0.02 |
| Ischemic stroke | 255 (7.0) | 252 (6.9) | 1.002 (0.84–1.19) | 0.98 |
| Acute myocardial infarction | 235 (6.5) | 216 (5.9) | 1.08 (0.90–1.30) | 0.41 |
| Composite of cardiovascular death, IS or AMI | 1,086 (29.8) | 1,162 (31.9) | 0.90 (0.83–0.98) | 0.02 |
| Heart failure hospitalization | 249 (6.8) | 219 (6.0) | 1.14 (0.95–1.36) | 0.17 |
| All-cause readmission | 2,801 (76.9) | 2,803 (77.0) | 0.977 (0.93–1.03) | 0.38 |
| Major adverse limb events | | | | |
| Newly-onset claudication | 50 (1.4) | 65 (1.8) | 0.76 (0.53–1.10) | 0.15 |
| Newly-onset critical limb ischemia | 222 (6.1) | 251 (6.9) | 0.87 (0.72–1.04) | 0.12 |
| Endovascular therapy | 180 (4.9) | 226 (6.2) | 0.78 (0.64–0.95) | 0.02 |
| Non-traumatic amputation event | 213 (5.8) | 280 (7.7) | 0.74 (0.62–0.89) | 0.001 |
| Composite outcome of EVT and amputation | 312 (8.6) | 392 (10.8) | 0.78 (0.67–0.90) | 0.001 |

HR, hazard ratio; SHR, subdistribution hazard ratio; CI, confidence interval;

Data were presented as frequency (percentage).

© 2022 Lo HY et al. *JAMA Network Open*.

**eTable 3.** Sensitivity Analysis Treating Different Statin Potency Levels as Time-Varying Exposures to Assess Association With the Risk of All-Cause Death and the Composite Outcome of Endovascular Therapy and Amputation in the Whole Cohort

| | No. of | Person- | Crude incidence | Unadjusted analysis | | | Adjusted analysis* | |
|---|---|---|---|---|---|---|---|---|
| Outcome/ period of exposure to statin | event | years | (95% CI)† | HR (95% CI) | *P* trend | | HR (95% CI) | *P* trend |
| All-cause mortality | | | | | <0.001 | | | <0.001 |
| Non-statin period | 2,699 | 14348.3 | 18.8 (11.7–25.9) | Reference | | | Reference | |
| Low to moderate potency | 1,276 | 9793.8 | 13.0 (5.9–20.2) | 0.68 (0.64–0.73) | | | 0.77 (0.72–0.83) | |
| High potency | 26 | 617.9 | 4.2 (0–20.4) | 0.22 (0.15–0.32) | | | 0.26 (0.18–0.39) | |
| Composite outcome of EVT and amputation | | | | | 0.830 | | | 0.110 |
| Non-statin period | 617 | 13888.1 | 4.4 (0.9–7.9) | Reference | | | Reference | |
| Low to moderate potency | 428 | 9411.6 | 4.5 (0.2–8.9) | 1.00 (0.89–1.13) | | | 0.90 (0.78–1.03) | |
| High potency | 31 | 597.6 | 5.2 (0–23.4) | 1.08 (0.76–1.54) | | | 0.85 (0.58–1.25) | |

HR, hazard ratio; CI, confidence interval; EVT, endovascular therapy;

† Number of events per 100 person-years;

* All of the baseline characteristics (listed in Table 1) were adjusted in the analysis, where the follow-up year was replaced by the index date.

© 2022 Lo HY et al. *JAMA Network Open*.

**eFigure 1.** Subgroup Analysis of the Association of Statin Therapy With the Risk of All-Cause Death and the Composite Outcome of Endovascular Therapy and Amputation in the Propensity Score–Matched Cohort

A. All-cause mortality

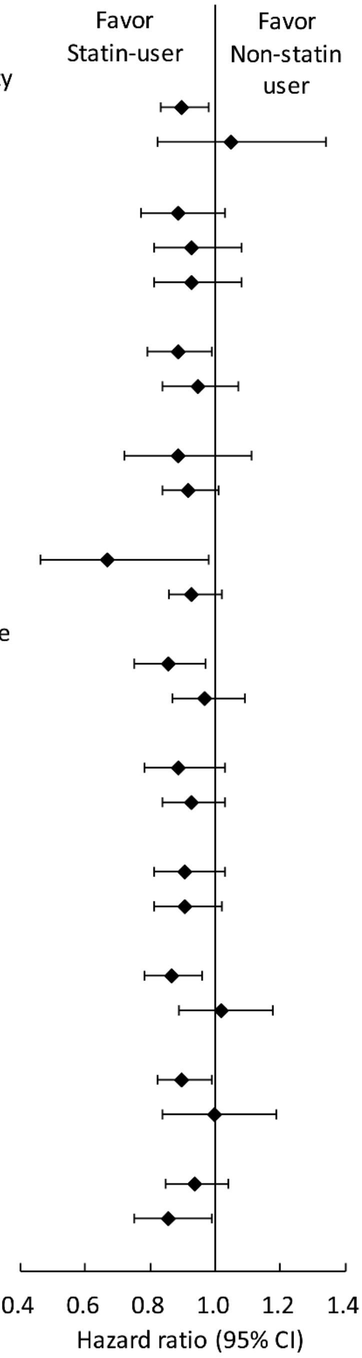


| | Event (%) Statin-user | Non-statin user | HR (95% CI) of Statin-user | *P* value for interaction |
|---|---|---|---|---|
| Initial dialysis modality | | | | 0.236 |
| HD | 952 (32.6) | 1,023 (35.1) | 0.90 (0.83–0.98) | |
| PD | 126 (40.1) | 115 (36.2) | 1.05 (0.82–1.34) | |
| Age | | | | 0.889 |
| 20 – 64 years | 338 (23.7) | 360 (25.7) | 0.89 (0.77–1.03) | |
| 65 – 74 years | 373 (35.2) | 397 (36.9) | 0.93 (0.81–1.08) | |
| ≥75 years | 367 (48.9) | 381 (50.3) | 0.93 (0.81–1.08) | |
| Sex | | | | 0.428 |
| Female | 572 (33.9) | 606 (36.3) | 0.89 (0.79–0.99) | |
| Male | 506 (32.7) | 532 (34.0) | 0.95 (0.84–1.07) | |
| Diabetes | | | | 0.783 |
| No | 153 (28.7) | 159 (31.0) | 0.89 (0.72–1.11) | |
| Yes | 925 (34.2) | 979 (36.0) | 0.92 (0.84–1.01) | |
| Hypertension | | | | 0.092 |
| No | 49 (27.2) | 60 (35.9) | 0.67 (0.46–0.98) | |
| Yes | 1,029 (33.7) | 1,078 (35.1) | 0.93 (0.86–1.02) | |
| Ischemic heart disease | | | | 0.134 |
| No | 466 (27.3) | 521 (30.3) | 0.86 (0.75–0.97) | |
| Yes | 612 (40.1) | 617 (40.7) | 0.97 (0.87–1.09) | |
| CCI total score | | | | 0.655 |
| ≤5 | 357 (27.3) | 380 (29.0) | 0.89 (0.78–1.03) | |
| >5 | 721 (37.4) | 758 (39.4) | 0.93 (0.84–1.03) | |
| Heart failure | | | | 0.984 |
| No | 544 (27.8) | 587 (29.7) | 0.91 (0.81–1.03) | |
| Yes | 534 (41.7) | 551 (43.8) | 0.91 (0.81–1.02) | |
| Ischemic stroke | | | | 0.063 |
| No | 686 (29.9) | 749 (32.8) | 0.87 (0.78–0.96) | |
| Yes | 392 (41.8) | 389 (41.0) | 1.02 (0.89–1.18) | |
| Myocardial infarction | | | | 0.296 |
| No | 838 (30.7) | 899 (33.0) | 0.90 (0.82–0.99) | |
| Yes | 240 (47.9) | 239 (46.6) | 1.00 (0.84–1.19) | |
| Previous LEAD | | | | 0.361 |
| No | 728 (31.2) | 767 (32.5) | 0.94 (0.85–1.04) | |
| Yes | 350 (38.9) | 371 (42.5) | 0.86 (0.75–0.996) | |

HD, hemodialysis; PD, peritoneal dialysis; CCI, Charlson’s Comorbidity Index; LEAD, lower extremity atrial disease; EVT, endovascular therapy

© 2022 Lo HY et al. *JAMA Network Open*.

B. Composite outcome of EVT and amputation

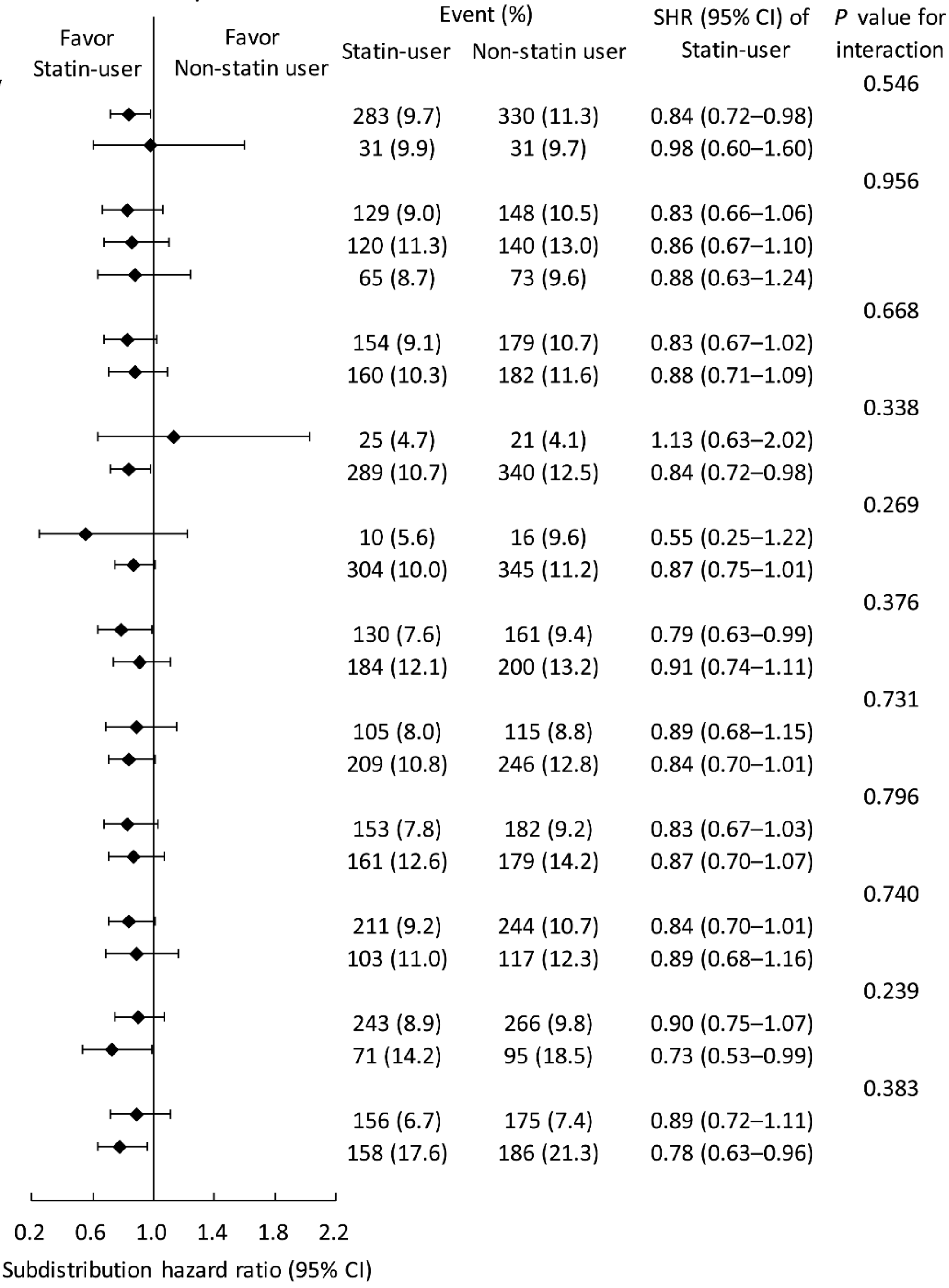


| | Event (%) Statin-user | Event (%) Non-statin user | SHR (95% CI) of Statin-user | *P* value for interaction |
|---|---|---|---|---|
| Initial dialysis modality | | | | 0.546 |
| HD | 283 (9.7) | 330 (11.3) | 0.84 (0.72–0.98) | |
| PD | 31 (9.9) | 31 (9.7) | 0.98 (0.60–1.60) | |
| Age | | | | 0.956 |
| 20 – 64 years | 129 (9.0) | 148 (10.5) | 0.83 (0.66–1.06) | |
| 65 – 74 years | 120 (11.3) | 140 (13.0) | 0.86 (0.67–1.10) | |
| ≥75 years | 65 (8.7) | 73 (9.6) | 0.88 (0.63–1.24) | |
| Sex | | | | 0.668 |
| Female | 154 (9.1) | 179 (10.7) | 0.83 (0.67–1.02) | |
| Male | 160 (10.3) | 182 (11.6) | 0.88 (0.71–1.09) | |
| Diabetes | | | | 0.338 |
| No | 25 (4.7) | 21 (4.1) | 1.13 (0.63–2.02) | |
| Yes | 289 (10.7) | 340 (12.5) | 0.84 (0.72–0.98) | |
| Hypertension | | | | 0.269 |
| No | 10 (5.6) | 16 (9.6) | 0.55 (0.25–1.22) | |
| Yes | 304 (10.0) | 345 (11.2) | 0.87 (0.75–1.01) | |
| Ischemic heart disease | | | | 0.376 |
| No | 130 (7.6) | 161 (9.4) | 0.79 (0.63–0.99) | |
| Yes | 184 (12.1) | 200 (13.2) | 0.91 (0.74–1.11) | |
| CCI total score | | | | 0.731 |
| ≤5 | 105 (8.0) | 115 (8.8) | 0.89 (0.68–1.15) | |
| >5 | 209 (10.8) | 246 (12.8) | 0.84 (0.70–1.01) | |
| Heart failure | | | | 0.796 |
| No | 153 (7.8) | 182 (9.2) | 0.83 (0.67–1.03) | |
| Yes | 161 (12.6) | 179 (14.2) | 0.87 (0.70–1.07) | |
| Ischemic stroke | | | | 0.740 |
| No | 211 (9.2) | 244 (10.7) | 0.84 (0.70–1.01) | |
| Yes | 103 (11.0) | 117 (12.3) | 0.89 (0.68–1.16) | |
| Myocardial infarction | | | | 0.239 |
| No | 243 (8.9) | 266 (9.8) | 0.90 (0.75–1.07) | |
| Yes | 71 (14.2) | 95 (18.5) | 0.73 (0.53–0.99) | |
| Previous LEAD | | | | 0.383 |
| No | 156 (6.7) | 175 (7.4) | 0.89 (0.72–1.11) | |
| Yes | 158 (17.6) | 186 (21.3) | 0.78 (0.63–0.96) | |

HD, hemodialysis; PD, peritoneal dialysis; CCI, Charlson's Comorbidity Index; LEAD, lower extremity atrial disease; EVT, endovascular therapy

© 2022 Lo HY et al. *JAMA Network Open*.

**eFigure 2.** Adjusted One-Minus Survival Rates of All-Cause Death and Fitted Cumulative Incidence Functions of the Composite Outcome of Endovascular Therapy and Amputation According to Different Statin Potency Levels

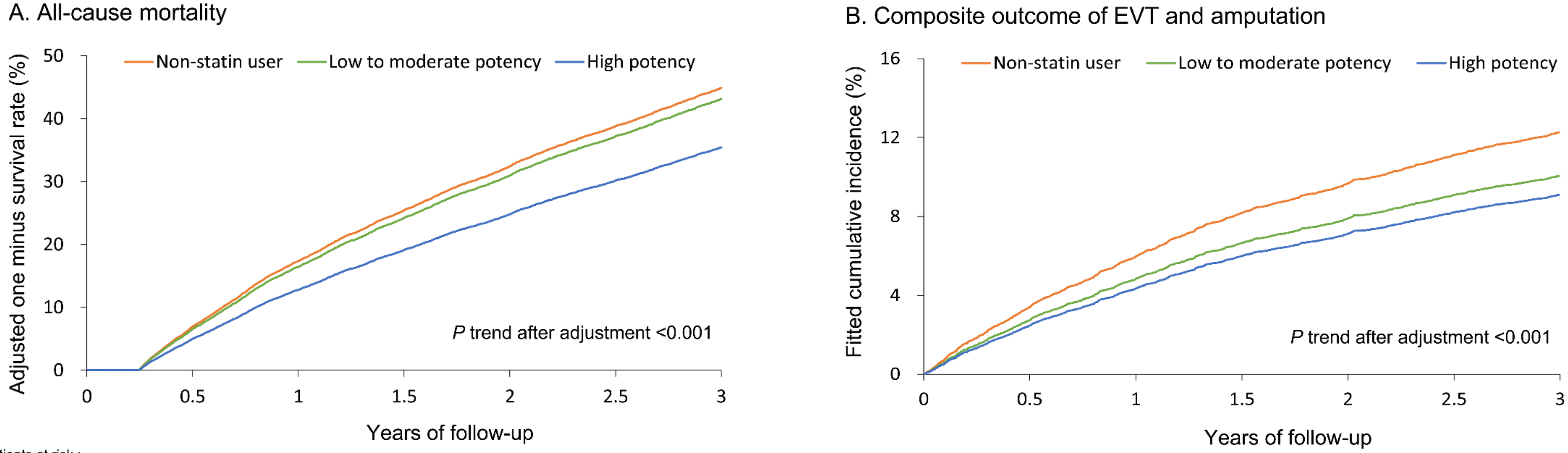


A. All-cause mortality — No. of patients at risk :

| | 0 | 0.5 | 1 | 1.5 | 2 | 2.5 | 3 |
|---|---|---|---|---|---|---|---|
| Non-statin | 7,170 | 6,412 | 5,291 | 4,462 | 3,749 | 3,190 | 2,656 |
| Low to moderate | 3,009 | 2,800 | 2,365 | 2,016 | 1,730 | 1,472 | 1,204 |
| High | 588 | 540 | 440 | 367 | 303 | 247 | 192 |

B. Composite outcome of EVT and amputation — No. of patients at risk :

| | 0 | 0.5 | 1 | 1.5 | 2 | 2.5 | 3 |
|---|---|---|---|---|---|---|---|
| Non-statin | 7,170 | 6,210 | 5,003 | 4,132 | 3,425 | 2,880 | 2,371 |
| Low to moderate | 3,009 | 2,725 | 2,259 | 1,881 | 1,589 | 1,336 | 1,084 |
| High | 588 | 527 | 420 | 349 | 284 | 228 | 176 |

EVT, endovascular therapy

© 2022 Lo HY et al. *JAMA Network Open*.
